# Altered systemic bioenergetic reserve in chronic kidney disease predisposes hearts to worse functional outcomes

**DOI:** 10.1038/s41598-025-25314-8

**Published:** 2025-11-21

**Authors:** Megan Young, Malene Aastrup, Nikayla Patel, Fenn Cullen, Esben S. S. Hansen, James E. Clark, Thomas R. Eykyn, Michael Væggemose, Ana Vujic, Loucia Karatzia, Ladislav Valkovič, Jack J. J. J. Miller, Niels H. Buus, Christoffer Laustsen, Magdi M. Yaqoob, Dunja Aksentijevic

**Affiliations:** 1 Barts and the London Faculty of Medicine and Dentistry, William Harvey Research Institute, Queen Mary University of London, Charterhouse Square, London, EC1M 6BQ UK; 2 The MR Research Centre, Department of Clinical Medicine, Aarhus University, Aarhus, Denmark; 3 The Rayne Institute, School of Cardiovascular and Metabolic Medicine and Sciences, Faculty of Life Sciences & Medicine, King’s College London, London, UK; 4 King’s College London BHF Centre of Research Excellence, London, UK; 5 Department of Imaging Chemistry and Biology, School of Biomedical Engineering and Imaging Sciences, King’s College London, London, UK; 6 GE HealthCare, Brondby, Denmark; 7 Department of Medicine, University of Cambridge, Cambridge, UK; 8 Oxford Centre for Clinical Magnetic Resonance Research (OCMR), University of Oxford, Oxford, UK; 9 Department of Imaging Methods, Institute of Measurement Science, Slovak Academy of Sciences, Bratislava, Slovakia; 10 Department of Renal Medicine, Aarhus University, Aarhus, Denmark

**Keywords:** Chronic kidney disease, Heart failure, Cardiomyopathies

## Abstract

Chronic kidney disease (CKD) is characterised by progressive loss of kidney function and structural damage, which contributes to systemic complications, including cardiovascular dysfunction. Inter-organ metabolic interactions are increasingly recognised as important in the pathophysiology of CKD, but the extent to which systemic bioenergetic deficits contribute to cardiac dysfunction remains unclear. We investigated cardiac and systemic metabolic remodeling in two rat models of CKD with distinct aetiologies: glomerulosclerosis induced by partial nephrectomy and interstitial fibrosis induced by an adenine-rich diet. Despite differing renal pathology, both models exhibited comparable cardiac dysfunction, including impaired recovery following 25 min of ischaemia. ^1^H NMR spectroscopy metabolomic analysis revealed that systemic metabolic alterations in skeletal muscle, liver, and kidney were more pronounced than those in the heart, indicating reduced systemic bioenergetic reserve. These findings were supported by data from CKD patients, in whom ^31^P NMR spectroscopy of exercising skeletal muscle demonstrated impaired phosphocreatine recovery, consistent with diminished bioenergetic capacity and reduced force generation. These results suggest that systemic bioenergetic impairment contributes to CKD-associated cardiac dysfunction. Targeting systemic metabolic derangements may represent a novel strategy to improve cardiac outcomes in CKD.

**Supplementary Information:**

The online version contains supplementary material available at 10.1038/s41598-025-25314-8.

## Introduction

Chronic kidney disease (CKD) affects 840 million people globally and is a leading cause of morbidity and mortality^1^. CKD is a progressive condition marked by structural kidney damage and declining renal function^2^. Beyond its direct effects on the kidneys, CKD is increasingly recognised as a systemic disorder that contributes to a range of complications, including cardiovascular disease, skeletal muscle wasting, and metabolic disturbances^3^.

Nevertheless, for many CKD patients the risk of developing cardiovascular disease is higher than progression to kidney failure^4^ with cardiovascular diseases remaining the leading cause of mortality. CKD cardiomyopathy is a distinct pathology characterised by diastolic dysfunction, left ventricular hypertrophy (LVH), fibrosis and maladaptive changes in cardiac metabolism^5,6^. In severe CKD, cardiac complications can progress into heart failure (HF), largely with preserved ejection fraction (HFpEF)^5,6^. While factors such as hypertension, volume overload, and uremic toxins are known contributors, the underlying mechanisms remain incompletely understood.

Emerging evidence suggests that inter-organ communication and systemic metabolic stress may play a critical role in CKD-related cardiac dysfunction. Skeletal muscle and liver, for example, are metabolically active tissues that undergo significant alterations in CKD and may contribute to or reflect systemic bioenergetic imbalance^7–9^. However, whether this systemic bioenergetic disturbance directly contributes to cardiac vulnerability has not been fully elucidated.

To address this gap, we investigated the relationship between systemic metabolic remodeling and cardiac dysfunction using two preclinical models of CKD with distinct aetiologies: glomerulosclerosis induced by partial nephrectomy and interstitial fibrosis induced by adenine feeding. This approach allowed us to assess whether a common systemic metabolic signature exists across models and whether it correlates with impaired cardiac performance. In parallel, we examined bioenergetic capacity in skeletal muscle from human CKD patients using ^31^P magnetic resonance spectroscopy to determine whether comparable bioenergetic deficiencies occur in clinical settings. Together, our study aims to clarify the role of systemic bioenergetic dysfunction in the pathogenesis of CKD-associated cardiac impairment and to identify potential metabolic targets for intervention.

## Materials and methods

### Animals

250 g male Wistar rats (*n* = 46) were purchased from Charles River Laboratories (UK). All animal experiments were approved by the Queen Mary University of London local ethical review committee and in accordance with the UK Home Office Animals (Scientific Procedures) Act, 1986 and ARRIVE guidelines. All animals were humanely sacrificed by pentobarbital I.P. injection.

### Experimental models of chronic kidney disease

Partial nephrectomy (PN) CKD was induced using an established two-stage surgical procedure for a period of 12 weeks (PN *n* = 13, Sham *n* = 9)^10–12^. Adenine diet model of CKD was induced by 4 week adenine dietary protocol (0.75% adenine in standard rat chow, Lillico Biotechnology) followed by 4 week standard chow diet (*n* = 15) with controls fed standard diet throughout (*n* = 9)^12,13^.

### Transthoracic echocardiography

In vivo cardiac function was assessed by echocardiography (Vevo 770;Visualsonics, Supplementary Materials).

### Biochemical analysis

Tissues (liver, skeletal muscle, heart, kidney) were rapidly collected upon termination and snap frozen in liquid N_2_^14^. Metabolomic profile was analysed using ^1^H NMR spectroscopy^14,15^. Plasma obtained from thoracic cavity blood samples taken at the time of sacrifice were analysed by the Clinical Biochemistry Laboratory (Addenbrookes Hospital, Cambridge).

### qPCR

RNA was isolated (Cat:74704;Qiagen) and reverse transcribed (Cat:4374967;Applied Biosystems). qPCR reactions were run in triplicates using SYBR green (Cat:600892;Agilent) on QuantStudio 5 (Applied Biosystems). Gene expression displayed relative to 36B4. Full methods and primer sequences in Supplementary Materials (Table S1).

### Langendorff heart perfusions

Hearts were rapidly excised, cannulated and perfused in Langendorff mode as previously described^16,17^. After 20 min of equilibration, hearts were subject to 25 min global normothermic ischaemia (37 °C) and 25 min reperfusion.

### Assessment of the human tibialis anterior muscle bioenergetic reserve by dynamic ^31^P-MRS

The clinical study protocol was approved by the Ethics Committee of Central Denmark (ref. 1-10-72-145-23) and all methods were performed in accordance with their guidelines and regulations. All subjects provided written informed consent prior to participation. Ten patients with non-diabetic CKD stage 3b and 4 and five age-matched healthy volunteers with normal renal function (Exclusion criteria in Supplementary Material) were recruited for ^31^P dynamic MRS scanning of the tibialis anterior muscle during exercise (Fig. S1)^18^. Scans were acquired at a 2 min resting baseline prior to 30 s of exercise followed by 10 min recovery. Full methods can be found in Supplementary Materials.

### Statistics

Statistical analysis was performed using GraphPad Prism software (version 10.1.0). Normality of data distribution was examined. Comparison between two groups was performed by Student’s t-test, with Welch’s correction for unequal variance applied accordingly, or Mann-Whitney U test used when data were non-normally distributed. A two-way ANOVA was used when two factors were present with post-hoc analysis completed if significant interaction present. Significance accepted when *p* < 0.05.

## Results

### CKD phenotype and cardiometabolic remodelling in CKD animal models

#### Adenine diet induced CKD

**Fig. 1:**
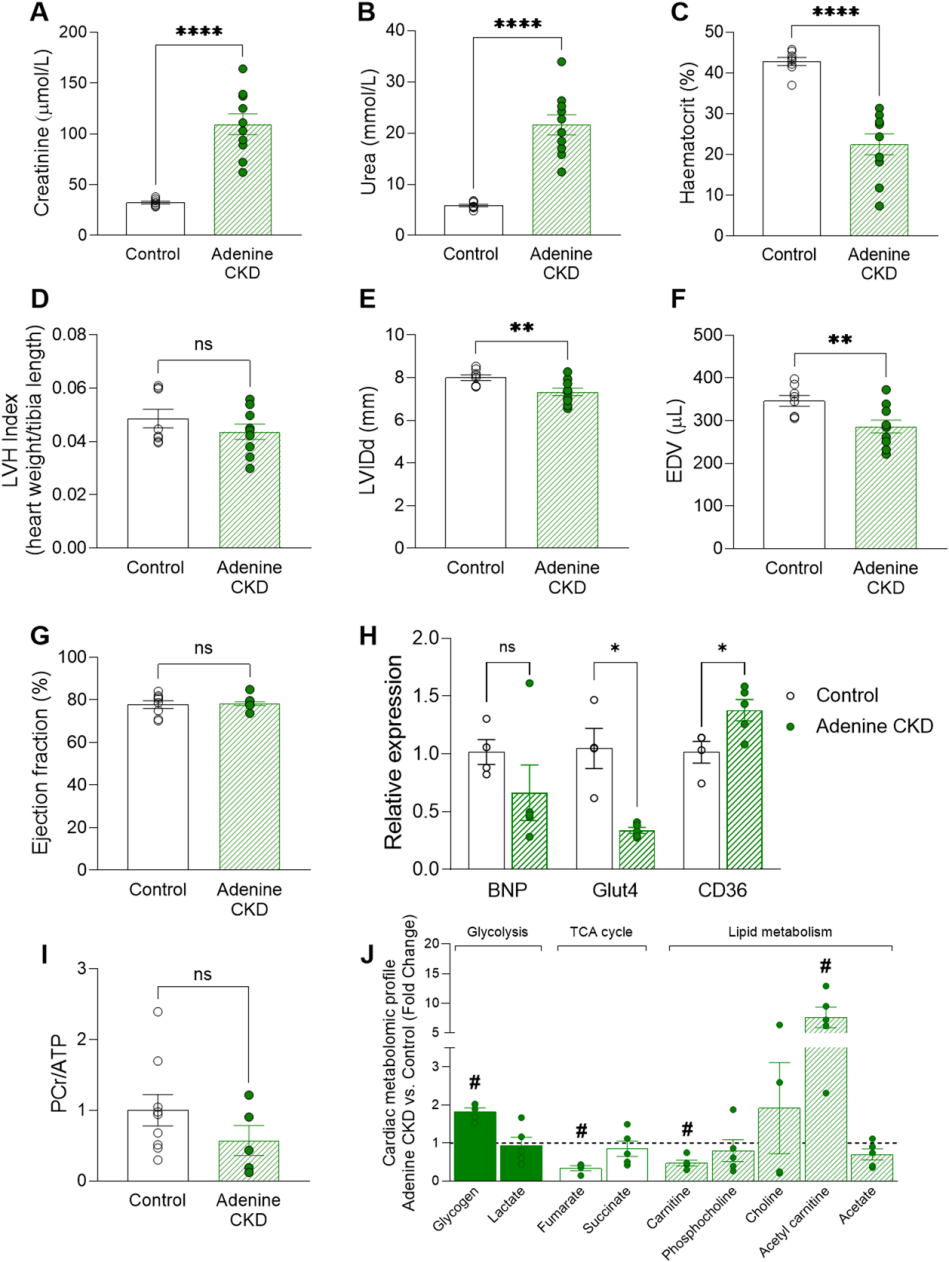
Morphological and cardiac metabolic changes in adenine diet CKD model. (**A, B**) Plasma levels of creatinine and urea (Adenine *n* = 10, control *n* = 8, unequal variance t-test, *p < 0.05). (**C**) Haematocrit measurement (Adenine *n* = 10, control *n* = 8, unequal variance t-test, *p < 0.05). (**D**) ratio of heart weight to tibia length (Adenine = 9, control *n* = 8, Student’s t-test, *p < 0.05), (**E–G**) Echocardiography measurement of left ventricle internal diameter in diastole (LVID, d), end diastolic volume (EDV) and ejection fraction (Adenine = 10, control *n* = 8, Student’s t-test, *p < 0.05). (**H**) mRNA expression of brain natriuretic peptide (BNP), glucose transporter 4 (Glut4) and fatty-acid transporter CD36 relative to housekeeping gene 36B4 (Adenine *n* = 5, control *n* = 4. BNP analysed by Mann-Whitney U test, Glut4 analysed by unequal variance t-test and CD36 analysed by Student’s t-test, (*p < 0.05). (**I**) Ratio of PCr/ATP measured by ^1^H NMR spectroscopy in cardiac tissue from adenine CKD (*n* = 5) and control diet (*n* = 9) animals, displayed as fold change. Analysed by Student’s t-test, *p < 0.05. (**J**) ^1^H NMR spectroscopy of cardiac tissue from adenine CKD (*n* = 5) displayed as fold change vs. control diet (*n* = 9) group. Statistical analysis displayed was performed using Student’s t-test comparison for each metabolite on untransformed metabolite levels (#*p* < 0.05); results and values are displayed in Table S4. Data displayed as mean ± SEM. (I-J) Metabolomic data displayed were acquired by ^1^H NMR spectroscopy using the aqueous phase containing water-soluble metabolites from a methanol/chloroform/water extraction protocol. Abbreviations: CKD, chronic kidney disease; PCr, phosphocreatine; ATP, adenosine triphosphate.

Adenine diet CKD induction protocol in Wistar rats led to renal dysfunction evident from increased plasma levels of creatinine and urea compared to control diet (Fig. 1A,B) as well as anaemia (Fig. 1C). Given the adenine CKD model is characterised by the absence of hypertension^12^, there was no increase in LVH index (Fig. 1D) or increases in LV wall thickness (Table S2). Changes in left ventricular internal diameter in diastole (LVID, d) and end diastolic volume (EDV) (Fig. 1E,F) reflect mild ventricular remodelling and compensatory increases in contractility to maintain ejection fraction (Fig. 1G). There is no evidence of congestive HF and lung oedema in adenine CKD (Fig. S2A). mRNA expression of BNP (Fig. 1H) as well as PCr/ATP ratio (Fig. 1I) were comparable between the adenine CKD heart and controls. ^1^H NMR spectroscopy assessment of adenine CKD myocardial metabolome revealed perturbed metabolic intermediates related to lipid metabolism (carnitine, acetyl carnitine), glycogen (1.8-fold increase) and the TCA cycle (67% reduction in fumarate) compared to control hearts (Figs. 1J, S3, Table S4). Furthermore, adenine diet-induced CKD caused marked changes in cardiac lipid content (Fig. S4). Expression of fatty acid transporter CD36 was increased in adenine CKD hearts, alongside a decreased expression of insulin-dependent glucose transporter GLUT4 (Fig. 1H). In terms of plasma metabolic profile (Table 1), adenine diet CKD animals had reduced circulating levels of glucose and insulin compared to control.

**Table 1:** Plasma analysis of adenine diet-CKD and Chow diet controls.

	Control (*n* = 8)	Adenine CKD (*n* = 10)
Glucose (mmol/L)	13.20 ± 0.35	10.01 ± 0.36*
Sodium (mmol/L)	142.1 ± 0.44	140.7 ± 1.17
Potassium (mmol/L)	4.98 ± 0.21	6.58 ± 0.32*
Chloride (mmol/L)	95.13 ± 0.69	96.40 ± 1.62
Triglycerides (mmol/L)	1.27 ± 0.28	0.82 ± 0.07
Insulin (µg/L)	1.83 ± 0.24	0.54 ± 0.11*
Free Fatty Acids (µmol/L)	337.6 ± 62.21	224.2 ± 34.01
Alanine aminotransferase (U/L)	40.14 ± 2.48	39.89 ± 2.64
Alkaline Phosphatase (U/L)	99.00 ± 8.11	125.7 ± 8.99*

Analysed by Student’s t-test, *p < 0.05 vs. Control. Data displayed as mean ± SEM.

### Partial nephrectomy (PN) CKD

**Fig. 2:**
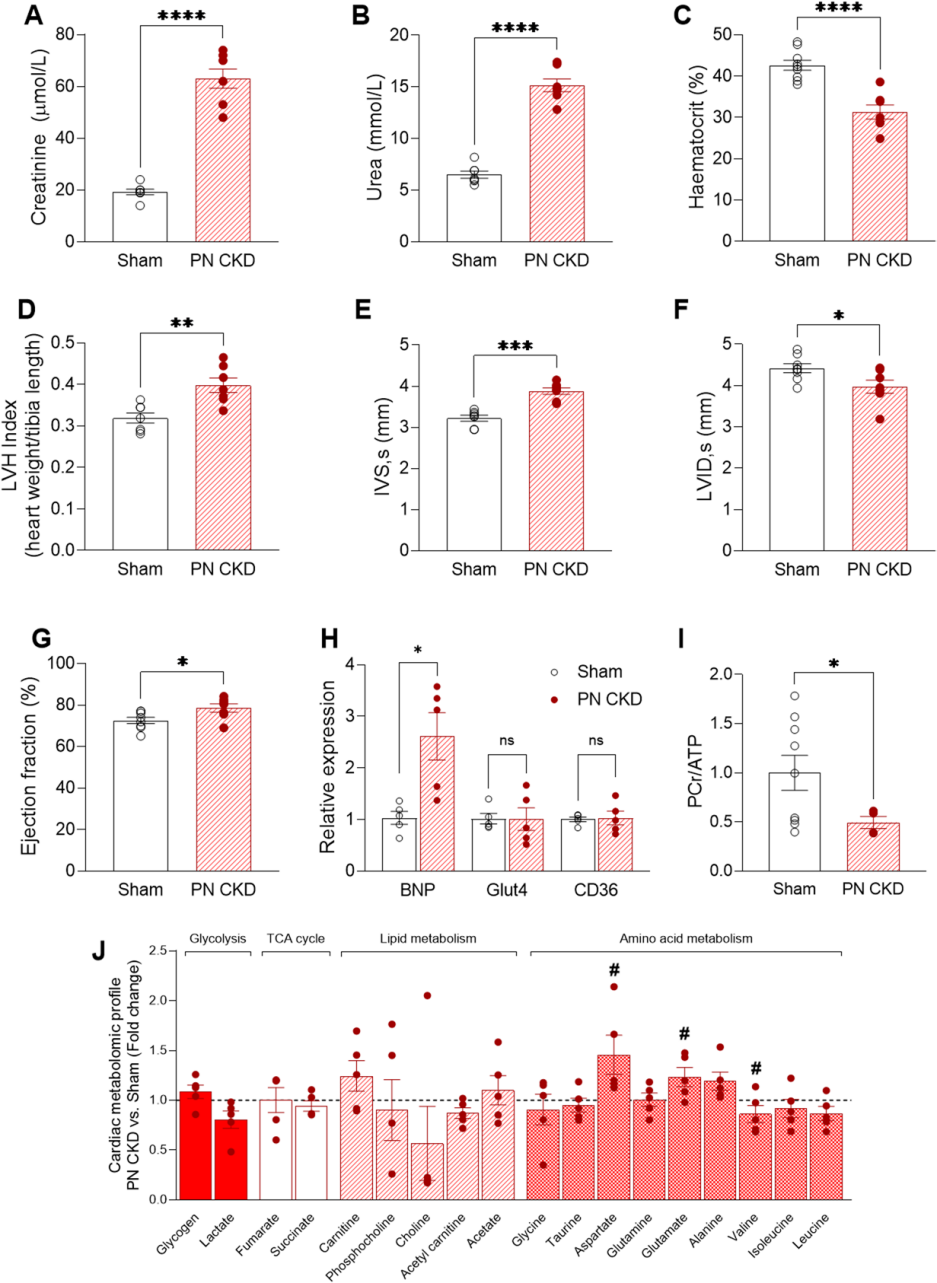
CKD phenotype and cardiometabolic remodelling in PN CKD model. (**A, B**) Plasma levels of creatinine and urea (PN CKD *n* = 7, sham *n* = 7, Student’s t-test, *p < 0.05). (**C**) Haematocrit measurement (PN CKD *n* = 7, sham *n* = 9, Student’s t-test, *p < 0.05). (**D**) ratio of heart weight to tibia length (PN CKD *n* = 7, sham *n* = 7, Student’s t-test, *p < 0.05. (**E-G**) Echocardiography measurement of intraventricular septum thickness in systole (IVS, s), left ventricle internal diameter in systole (LVID, s) and ejection fraction (PN CKD *n* = 7, sham *n* = 8, Student’s t-test, *p < 0.05). (**H**) mRNA expression of brain natriuretic peptide (BNP), glucose transporter 4 (Glut4) and fatty-acid transporter CD36 relative to housekeeping gene 36B4 (PN CKD *n* = 5, sham *n* = 5. BNP analysed by unequal variance t-test, Glut4 and CD36 analysed by Student’s *t*-test, *p < 0.05). (**I**) Ratio of PCr/ATP measured by ^1^H NMR spectroscopy in cardiac tissue from PN CKD (*n* = 4) and sham (*n* = 9) animals, displayed as fold change. Analysed by unequal variance t-test, *p < 0.05. (**J**) ^1^H NMR spectroscopy of cardiac tissue from PN CKD (*n* = 5) expressed as fold change vs. sham group (*n* = 9). Statistical analysis displayed was performed using Student’s t-test comparison for each metabolite on untransformed metabolite levels (#*p* < 0.05); results and values are displayed in Table S5. Data displayed as mean ± SEM. (**I**–**J**) Metabolomic data displayed were acquired by ^1^H NMR spectroscopy using the aqueous phase containing water-soluble metabolites from a methanol/chloroform/water extraction protocol. Abbreviations: PN, partial nephrectomy; CKD, chronic kidney disease; PCr, phosphocreatine; ATP, adenosine triphosphate.

Two-stage PN surgery induced a CKD phenotype characterised by renal dysfunction (elevated plasma levels of creatinine and urea—Fig. 2A,B), anaemia (Fig. 2C) and cardiometabolic remodelling. In addition to renal dysfunction and anaemia, hypertension is present in PN model of CKD from the onset^10,19,20^. 12-weeks of CKD progression led to pathological ventricular remodelling observed from an increase in LVH index (Fig. 2D). In vivo cardiac assessment identified changes in cardiac wall thickness and decreased internal diameter (Fig. 2E,F, Table S3). Furthermore, elevated mRNA expression of BNP (Fig. 2H) is indicative of developing HF however in the absence of a reduction in ejection fraction (Fig. 2G).

Assessment of circulating metabolites showed a decline in plasma glucose levels in PN CKD (Table 2). ^1^H NMR myocardial metabolomic profiling of PN CKD identified changes in amino acid metabolism (Fig. 2J, Table S5) without alterations in the expression of metabolic transporters (CD36 and Glut4, Fig. 2H). However, cardiac energy reserve was significantly impaired in PN CKD with a 50% reduction in PCr/ATP ratio compared to sham hearts (Fig. 2I). Comparison of the severity of remodelling in PN vs. adenine-diet induced CKD is summarised in Supplementary Figs. S5 and S6.

**Table 2:** Plasma analysis of PN CKD and Sham controls.

	Sham (*n* = 7)	PN CKD (*n* = 7)
Glucose (mmol/L)	8.3 ± 0.65	7.64 ± 0.27
Sodium (mmol/L)	138.9 ± 1.01	138.8 ± 0.4
Potassium (mmol/L)	4.87 ± 0.18	5.58 ± 0.39
Chloride (mmol/L)	92.43 ± 1.66	94.0 ± 0.72
Triglycerides (mmol/L)	1.5 ± 0.18	1.25 ± 0.21
Insulin (µg/L)	0.79 ± 0.44	0.44 ± 0.07
Free Fatty Acids (µmol/L)	790.1 ± 142	757.6 ± 156
Alanine aminotransferase (U/L)	43.43 ± 3.68	32.33 ± 2.41*
Alkaline Phosphatase (U/L)	85.57 ± 5.55	78.14 ± 7.13

Analysed by Student’s t-test, *p < 0.05 vs. Sham. Data displayed as mean ± SEM.

### CKD increases susceptibility to cardiac ischaemia reperfusion injury

**Fig. 3:**
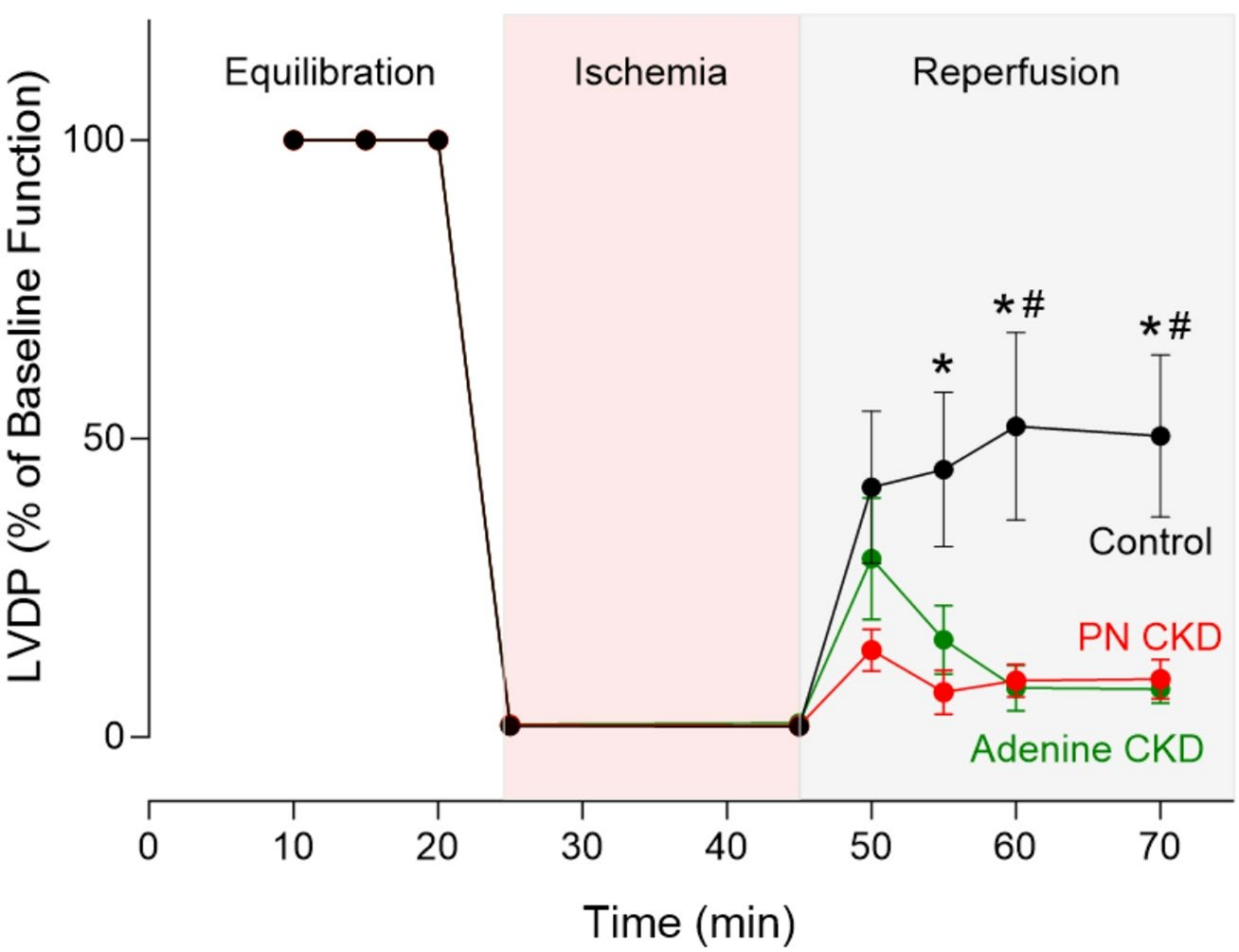
Ischaemia and reperfusion in CKD hearts. Functional recovery in PN CKD (*n* = 4), adenine CKD (*n* = 5) and control hearts (*n* = 5). Left ventricle developed pressure (LVDP) displayed as a percentage of baseline value. Analysed by two-way repeated measures ANOVA, significant interaction (*p* < 0.05) between groups over time. One-way ANOVA analysis for each time point displayed, *p < 0.05 PN CKD vs. control, #*p* < 0.05 Adenine CKD vs. control. Data displayed as mean ± SEM. Abbreviations: PN, partial nephrectomy; CKD, chronic kidney disease.

Given the evidence of altered cardiometabolic profile of CKD hearts, we examined the response to ischaemia and reperfusion. When stressed by a period of 25-min total global normothermic ischaemia followed by 25-min reperfusion, both adenine and PN CKD hearts display poor outcomes (Fig. 3). Given the unaltered PCr/ATP ratio, adenine CKD hearts do show signs of improved recovery during the immediate onset of reperfusion, however this is not sustained (Fig. 3). At the end of 25-min reperfusion, adenine and PN CKD hearts left ventricle developed pressure (LVDP) recovered 8.02% and 9.75% respectively compared to 50.42% recovery in control hearts (Fig. 3).

### CKD development leads to systemic metabolic alterations

#### Skeletal muscle

**Fig. 4:**
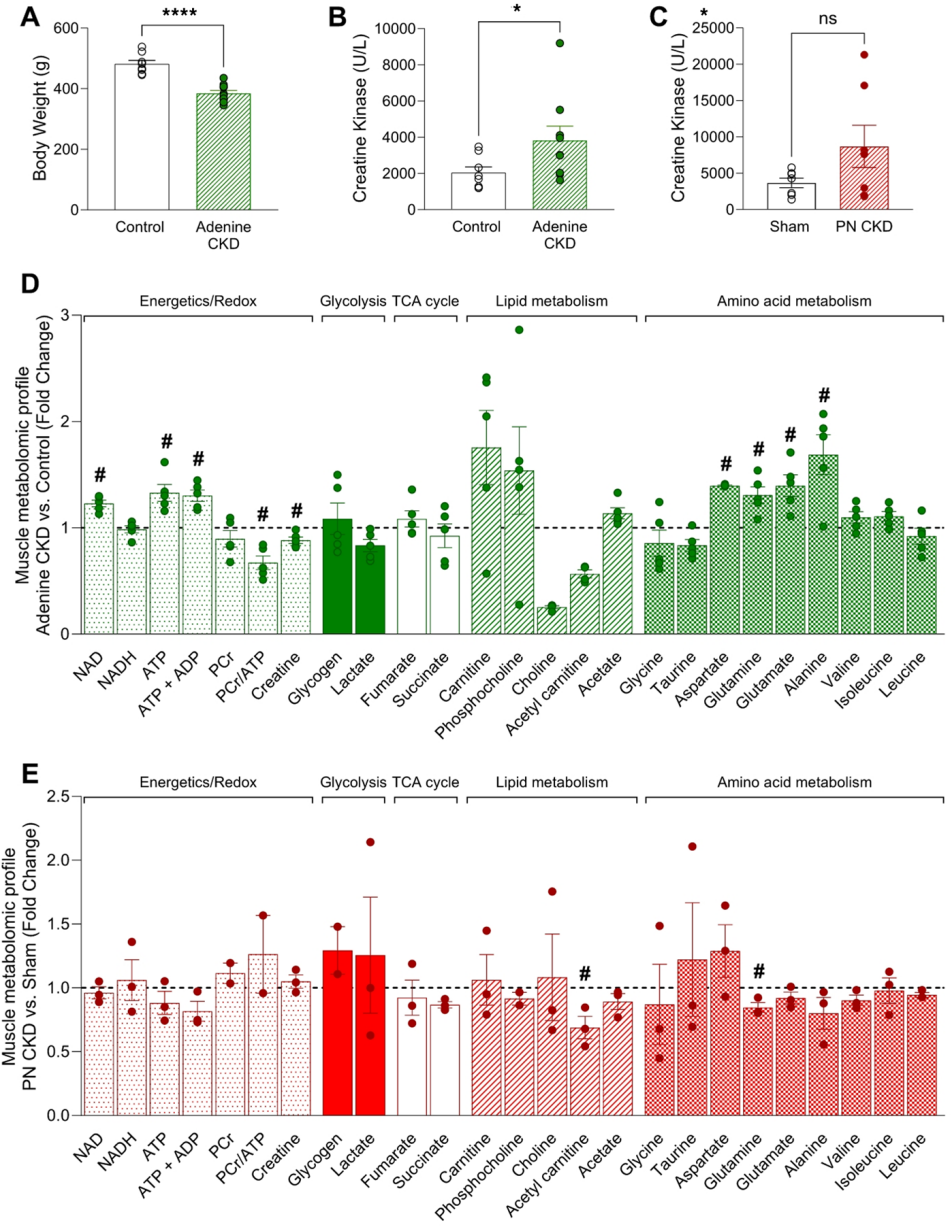
Metabolomic profile of skeletal muscle in adenine and PN CKD models. (**A**) Terminal body weight in adenine CKD animals. Analysed by Student’s t-test, *p < 0.05, control *n* = 8, Adenine *n* = 10 (**B,C**) Plasma creatine kinase level in (B) adenine diet CKD animals (Analysed by Mann-Whitney U test, *p < 0.05, control *n* = 8, Adenine *n* = 10) and (C) PN CKD animals (Analysed by unequal variance t-test, *p* > 0.05, sham *n* = 7, PN CKD *n* = 7). (**D, E**) Muscle metabolomic profile measured by ^1^H NMR spectroscopy of (D) adenine diet CKD (*n* = 5) vs. control diet (*n* = 5) and (E) PN CKD (*n* = 3) vs. sham (*n* = 3). Statistical analysis displayed was performed using Student’s t-test comparison for each metabolite on untransformed metabolite levels (#*p* < 0.05); results and values are displayed in Tables S6 and S7. Data displayed as mean ± SEM. Metabolomic data displayed were acquired by ^1^H NMR spectroscopy using the aqueous phase containing water-soluble metabolites from a methanol/chloroform/water extraction protocol. Abbreviations: PN, partial nephrectomy; CKD, chronic kidney disease; NAD, nicotinamide adenine dinucleotide; ATP, adenosine triphosphate; ADP, adenosine diphosphate; PCr, phosphocreatine.

Skeletal muscle damage was evident in adenine CKD animals and to a lesser extent in PN CKD. Adenine CKD was associated with increased circulating levels of creatine kinase (Fig. 4B), as well as a reduction in body weight (Fig. 4A) indicative of muscle wastage. The metabolomic profiling of skeletal muscle from adenine CKD animals revealed a decline in energy reserve with a 33% reduction in PCr/ATP ratio, accompanied by increased amino acids concentrations (aspartate, alanine, glutamine, glutamate) (Fig. 4D, Table S6). The PN CKD model was characterised by doubling of circulating creatine kinase (Fig. 4C), reduction in skeletal muscle acetyl carnitine (0.68-fold change) and changes in amino acid profile (Fig. 4E, Table S7).

#### Kidney

**Fig. 5:**
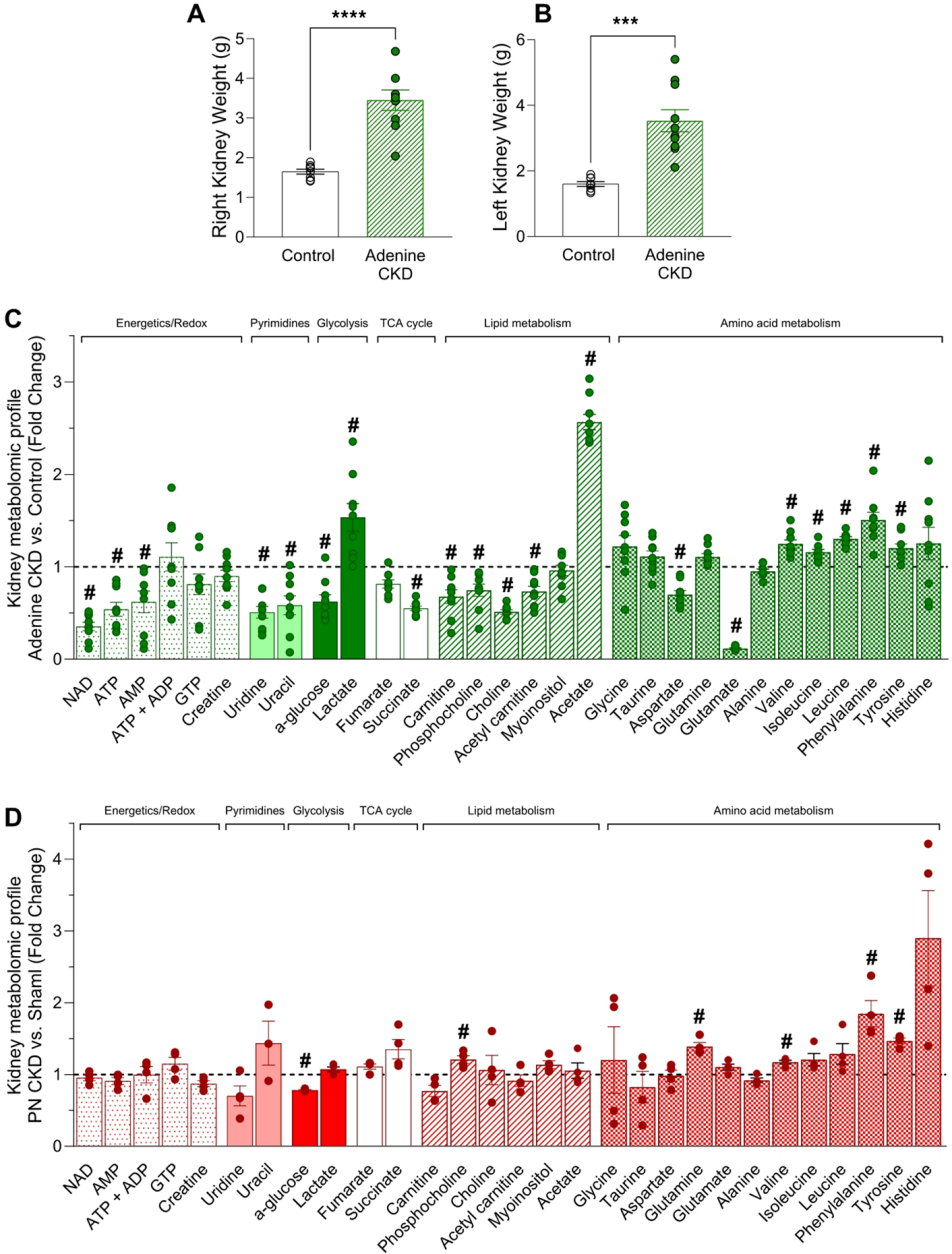
Kidney morphology and metabolomic profile of adenine and PN CKD models. (**A, B**) Right and left kidney weight. Analysed by unequal variance t-test, *p < 0.05, control *n* = 8, Adenine *n* = 10. (**(C, D**) Kidney metabolomic profile measured by ^1^H NMR spectroscopy of (D) adenine diet CKD (*n* = 9) vs. control diet (*n* = 5) and (E) PN CKD (*n* = 4) vs. sham (*n* = 4). Statistical analysis displayed was performed using Student’s t-test comparison for each metabolite on untransformed metabolite levels (#*p* < 0.05); results and values are displayed in Tables S8 and S9. Data displayed as mean ± SEM. Metabolomic data displayed were acquired by ^1^H NMR spectroscopy using the aqueous phase containing water-soluble metabolites from a methanol/chloroform/water extraction protocol. Abbreviations: PN, partial nephrectomy; CKD, chronic kidney disease; NAD, nicotinamide adenine dinucleotide; AMP, adenosine monophosphate; ATP, adenosine triphosphate; ADP, adenosine diphosphate; GTP, guanosine triphosphate.

Renal dysfunction is evident in both CKD models with increased plasma levels of creatinine and urea (Figs. 1A,B and 2A,B). In the adenine CKD model both kidneys are intact accompanied by significant kidney hypertrophy (Fig. 5A,B).

Kidney morphology is harder to compare with weights in the PN CKD model given the surgical resection, although there is visual evidence of compensatory hypertrophy in the 1/3 remaining kidney.

Both experimental models of CKD had marked changes in the metabolomic profile of kidney tissue, to a greater extent in the adenine model (Fig. 5C,D). In the adenine CKD model overt metabolic changes were observed with 20 changed out of 32 metabolites studied (Fig. 5C, Table S8). Both CKD models show increases in several amino acids in the kidney (Fig. 5C,D, Tables S8 and S9). While the magnitude of these changes differs, the overlap in directionality indicates that amino acid metabolism is commonly remodeled across both models.

#### Liver

**Fig. 6:**
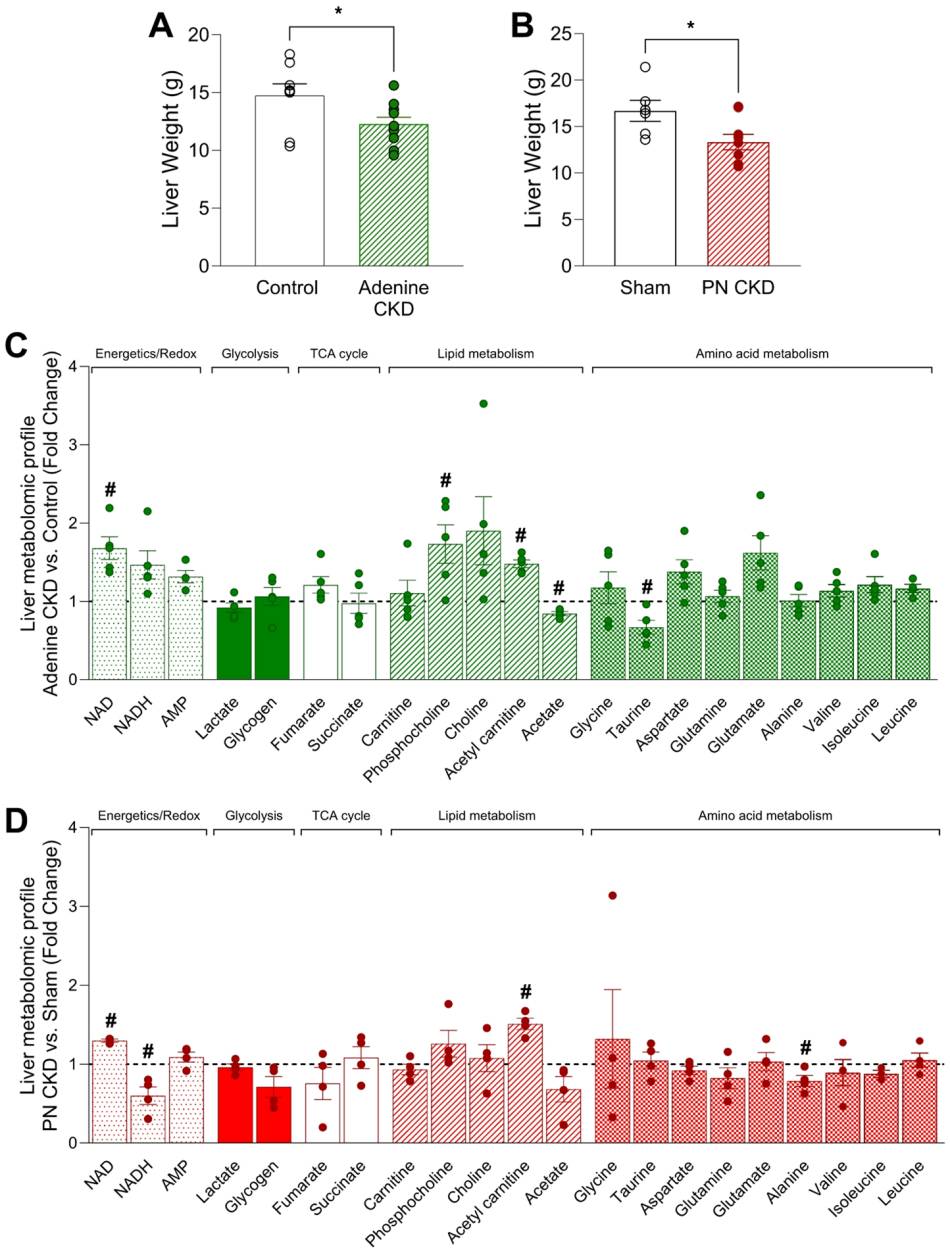
Metabolomic profile of liver in adenine and PN CKD models. (**A, B**) Liver weight in (A) adenine diet CKD (*n* = 10) vs. control diet (*n* = 8) animals and (B) PN CKD (*n* = 9) and sham (*n* = 6) animals. Analysed by Student’s t-test, *p < 0.05. (**C, D**) Liver metabolomic profile measured by ^1^H NMR spectroscopy of (C) adenine diet CKD (*n* = 5) vs. control diet (*n* = 4) and (D) PN CKD (*n* = 4) vs. sham (*n* = 4). Statistical analysis displayed was performed using Student’s t-test comparison for each metabolite on untransformed metabolite levels (#*p* < 0.05); results and values are displayed in Tables S10 and S11. Data displayed as mean ± SEM. Metabolomic data displayed were acquired by ^1^H NMR spectroscopy using the aqueous phase containing water-soluble metabolites from a methanol/chloroform/water extraction protocol. Abbreviations: PN, partial nephrectomy; CKD, chronic kidney disease; NAD, nicotinamide adenine dinucleotide; AMP, adenosine monophosphate.

The liver phenotype of both CKD models included reduction in liver weight (Fig. 6A,B). There were changes in the liver metabolomic profile of adenine CKD animals including a 33% reduction in taurine levels, and alterations in metabolites involved in lipid metabolism (Fig. 6C, Table S10). In PN CKD, hepatic acetyl carnitine was increased ~ 1.5-fold compared to sham, which is quantitatively comparable to the increase observed in the adenine model (Fig. 6D, Table S11).

### ^31^P MRS assessment of exercising leg muscle of CKD patients identifies functional bioenergetic impairment

**Fig. 7:**
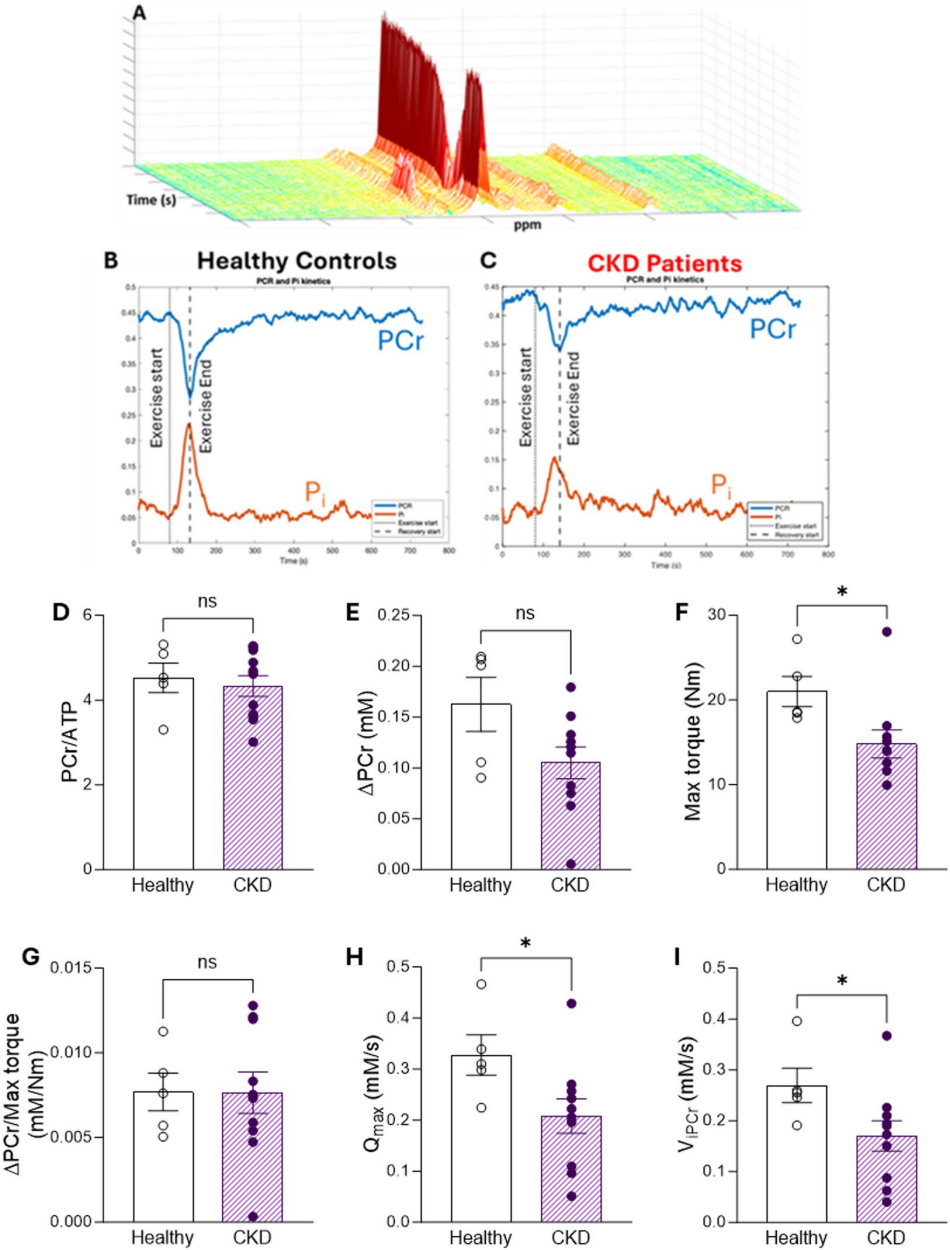
Human ^31^P MRS in tibialis anterior muscle of healthy controls and CKD patients during exercise. (**A**) Dynamic ^31^P spectra. (**B, C**) Change in PCr and Pi before, during and after 30 s of exercise. (**D**) Baseline PCr levels, (**E**) ΔPCr (difference in PCr between rest and end of exercise), (**F**) Maximum torque generation during exercise and (**G**) ΔPCr normalised to maximum torque generation in CKD patients and healthy controls. (**H**) Estimated mitochondrial capacity for maximal ATP resynthesis (Q_max_), determined as described in the text. (**I**) Estimated initial rate of PCr resynthesis following exercise as described in the test. (**D**–**I**) *p < 0.05, healthy controls *n* = 5, CKD patients *n* = 10. (**D**, **G**–**H**) Analysed by unequal variance *t*-test, (**E**–**F**, **I**) analysed by Mann-Whitney U test. Data displayed as mean ± SEM.

Given the finding of perturbed metabolism in the peripheral tissues of the CKD animal models, we sought to measure the bioenergetic reserve in skeletal muscle of CKD patients when challenged with exercise. The demographic and clinical characteristics of the CKD patients and healthy control study participants is shown in Table 3. ^31^P MRS was obtained from the tibialis anterior muscle at baseline, during exercise, and during the 10-min recovery period (Fig. 7A). The depletion of PCr can be observed across the 30-second exercise period, followed by resynthesis during recovery (Fig. 7B,C). At baseline, CKD patients had a comparable PCr/ATP ratio to healthy controls (Fig. 7D), however the utilisation of PCr during exercise was 35% lower in the CKD patient group (ΔPCr, Fig. 7E). Furthermore, we examined the maximum torque generation during exercise and found the CKD patient group generated significantly lower force than healthy controls (Fig. 7F).

**Table 3:** Demographics of CKD patients and healthy controls.

	Healthy controls, (*n* = 5)	*n*, number of values	CKD patients, (*n* = 10)	*n*, number of values
Age (years)	54 ± 12	5	61 ± 11.68	10
Gender, n (%)		5		10
- Male	4 (80%)		8 (80%)	
- Female	1 (20%)		2 (20%)	
Height (cm)	176 ± 8	5	174 ± 9	9
Weight (kg)	80 ± 14	5	88 ± 19	10
BMI (kg/m^2^)	26 ± 4	5	29 ± 2	9
Biochemistry of non-diabetic CKD patients* 45 − 30,** 30 − 15**,** < 15**				
eGFR (ml/min/1.73 m^2^)			26 ± 7	10
- eGFR 45 − 30			38	2
- eGFR 30 − 15			24 ± 7	8
UACR (mg/g)			422 ± 327	10
- UACR > 1000			1191	1
- UACR < 1000 − 500			524 ± 21	2
- UACR < 500 − 100			313 ± 186	6
- UACR < 100			96	1
**Medical history of non-diabetic CKD patients**				
Hypertension, n (%)				10
Yes**			9 (90%)	
No			1 (10%)	
Aetiology, n (%)				10
Hypertensive nephropathy			4 (40%)	
ANCA-associated vasculitis			2 (20%)	
Obstructive nephropathy			1 (10%)	
ADPKD			1 (10%)	
Sequelae after acute kidney failure (due to sepsis)			1 (10%)	
CKD of unknown cause			1 (10%)	
Stage of CKD, n (%)				10
CKD stage 3b			2 (20%)	
CKD stage 4			8 (80%)	
Duration of illness with eGFR < 60, n (%)				10
5–10 years			3 (30%)	
> 10 years			7 (70%)	
Medications				
RAS inhibitors			9	
Beta blockers			3	
Calcium blockers			4	
Diuretics			5	
SGLT2i			6	

Data displayed as mean ± SD. *Most recent values, **All treated with antihypertensive medication. Abbreviations: eGFR – estimated Glomerular Filtration Rate, UACR – Urine-Albumin-Creatinine-ratio, ANCA – anti-neutrophilocyte-cytoplasmic-antibody, ADPKD – autosomal dominant polycystic kidney disease, RAS – renin-angiotensin system, SGLT2i-sodium-glucose co-transporter 2 inhibitor.

Whilst the muscle PCr utilisation is comparable between CKD patients and controls when torque generation is accounted for (Fig. 7G), mitochondrial capacity (Qmax) is significantly decreased (Fig. 7H) alongside a decreased initial rate of PCr resynthesis (Fig. 7I). The overall PCr recovery indicated by Tau as well as pH was comparable between CKD patients and healthy controls (Fig. S7A,B).

## Discussion

Given the systemic nature of CKD and metabolic crosstalk between organs, we investigated both the cardiac and systemic metabolic signature (skeletal muscle, liver, kidney) of two CKD animal models with two distinct aetiologies, durations and severities: tubulointerstitial fibrosis and glomerulosclerosis. Moreover, we assessed the metabolic capacity of skeletal muscle of human CKD patients. This study identified that CKD, regardless of aetiology, leads to cardiometabolic remodelling and increased susceptibility to ischaemia reperfusion injury. However, changes in systemic metabolomic profile (muscle, liver, kidney) exceed severity of cardiac metabolic remodelling. We also show that despite comparable bioenergetic capacity to healthy controls at rest, CKD patients have an inability to utilise their muscle bioenergetic reserve. Therefore, our study shows inability of both heart and skeletal muscles in CKD to respond to a metabolic stress.

Adenine-diet induced CKD is of distinct aetiology to PN and induces CKD with the absence of hypertension^12^. However, cardiometabolic remodelling is less extensively reported for the adenine-diet induced CKD experimental model. The adenine CKD model caused a more severe perturbation of renal function and anaemia, as well as significantly impaired systemic glucose homeostasis (hypoglycaemia, hypoinsulinaemia) not seen in the PN CKD model. The absence of pressure overload is evidenced by the lack of LVH. Observed cardiac functional changes are suggestive of compensatory functional response to maintain ejection fraction under hyperdynamic confounders including severe anaemia.

Decline in kidney function impacts the reabsorption and therefore availability of metabolites within the body. Metabolic profile of adenine-diet induced CKD cardiomyopathy was characterised by reduced exogenous glucose supply due to circulating hypoglycaemia and hypoinsulinaemia which could be responsible for reduced expression of insulin-responsive myocardial glucose transporter GLUT4. Increased expression of fatty acid transporter CD36 accompanied by increased myocardial fatty acid metabolism intermediate (acetyl carnitine) are indicative of increased myocardial uptake and utilisation of fatty acids for the maintenance of cardiac energy reserve as PCr/ATP and glycogen content were maintained. Depleted myocardial lipid pools are also indicative of increased use of fatty acids. Given there is no energetic deficit and no increase in BNP mRNA expression, there is no evidence of HF in adenine diet-induced CKD.

We have previously shown that experimental CKD induced by PN leads to development of anaemia, hypertension, LVH and substrate utilisation switch from fatty acid to glucose utilisation concurrent with a decline in energy reserve^7,10,11,19–21^. Here we show that although there are no extensive changes in intermediate myocardial metabolite concentration in PN CKD, alterations in myocardial amino acid profile are indicative of metabolic adaptation to maintain ATP supply^17^. However, these metabolic adaptations were insufficient to maintain a healthy cardiac phenotype as reduced PCr/ATP and elevated BNP mRNA expression are indicative of progression into HF with a preservation of ejection fraction.

Any underlying cardiac metabolic adaptations in CKD may be sufficient to maintain bioenergetic reserve or cardiac function under basal conditions, but the ability to respond to stress is ultimately impaired. We found that irrespective of the renal failure aetiology and the state of energy reserve of the heart, the presence of CKD with its multifaceted pathophysiology enhances the myocardial susceptibility to ischaemic stress. Adenine CKD hearts show recovery during the initial 5 min of reperfusion reflective of the preserved baseline energetics but ultimately recovery during reperfusion is poor. This increased susceptibility to ischaemia reperfusion injury is in line with previously reported enhanced susceptibility to dobutamine stress, increased susceptibility to mitochondrial permeability transition pore opening in CKD models as well as increased incidence of ischaemic cardiac death in CKD patients^7,22–24^.

Muscle wastage, fatigue and poor physical performance are common in CKD, and a major debilitating feature impacting patients’ quality of life^25,26^. Changes in the adenine model CKD such as reduced body weight and increased circulating creatine kinase are indicative of muscle damage. We also found that a muscle specific decline in PCr/ATP ratio in the adenine diet-induced CKD model preceded decline in cardiac bioenergetic reserve. This suggests that in CKD, skeletal muscle bioenergetic impairment precedes detrimental cardiac changes. Previous work has shown skeletal muscle bioenergetic reserve correlates with cardiac bioenergetic decline in experimental CKD, highlighting the metabolic and functional relationship between organs^7^. We investigated this relationship further in CKD patients. We found that despite comparable baseline energy reserve, during exercise CKD patients were unable to utilise PCr to the same extent as healthy controls. Furthermore, torque generation was lower in CKD patients versus healthy controls. Given the relationship between CKD, muscular wastage and fatigue this may be reflected in reduced capacity for muscle force generation observed. Our findings are in line with previous reports that CKD patients have reduced peak oxygen capacity, skeletal muscle mitochondrial capacity, reduced mitochondrial number, mitochondrial dysfunction and increased superoxide generation^27–29^. Furthermore, in the calf muscle of haemodialysis patients, ATP supply via oxidative metabolism was shown to be impaired and compensated for by an increase in anaerobic glycolysis^30^. We found that the utilisation of PCr relative to the torque generation was comparable. Therefore, the bioenergetic impairment in skeletal muscle of CKD patients is not solely governed by the availability of PCr nor by a need to utilise greater amounts of energy to produce the same level of force. The limiting factor appears to be an inability to utilise the available energy reserve, leading to the development of fatigue.

Kidney metabolome was perturbed in both experimental models. Severe renal injury caused by adenine deposits in the kidney^12^ led to extensive metabolic alterations. Amongst the multiple systemic complications in CKD, our study also identified impact on the liver. Reduction in liver weight as well as increase in plasma liver function enzyme marker is indicative of chronic liver damage. Both CKD models are characterised by extensive alterations in liver redox, energy reserve and elevation of the lipid metabolism intermediates. Furthermore, a reduction in taurine was noted in liver from adenine CKD models, a key regulator of hepatic oxidative stress and fatty liver disease^31^. CKD is associated with higher prevalence of liver metabolic diseases such as cirrhosis and non-alcoholic fatty liver disease as two pathologies share several risk factors, including insulin resistance, oxidative stress, inflammation alongside an increased prevalence of cardiovascular disease^9,32,33^.

**Fig. 8:**
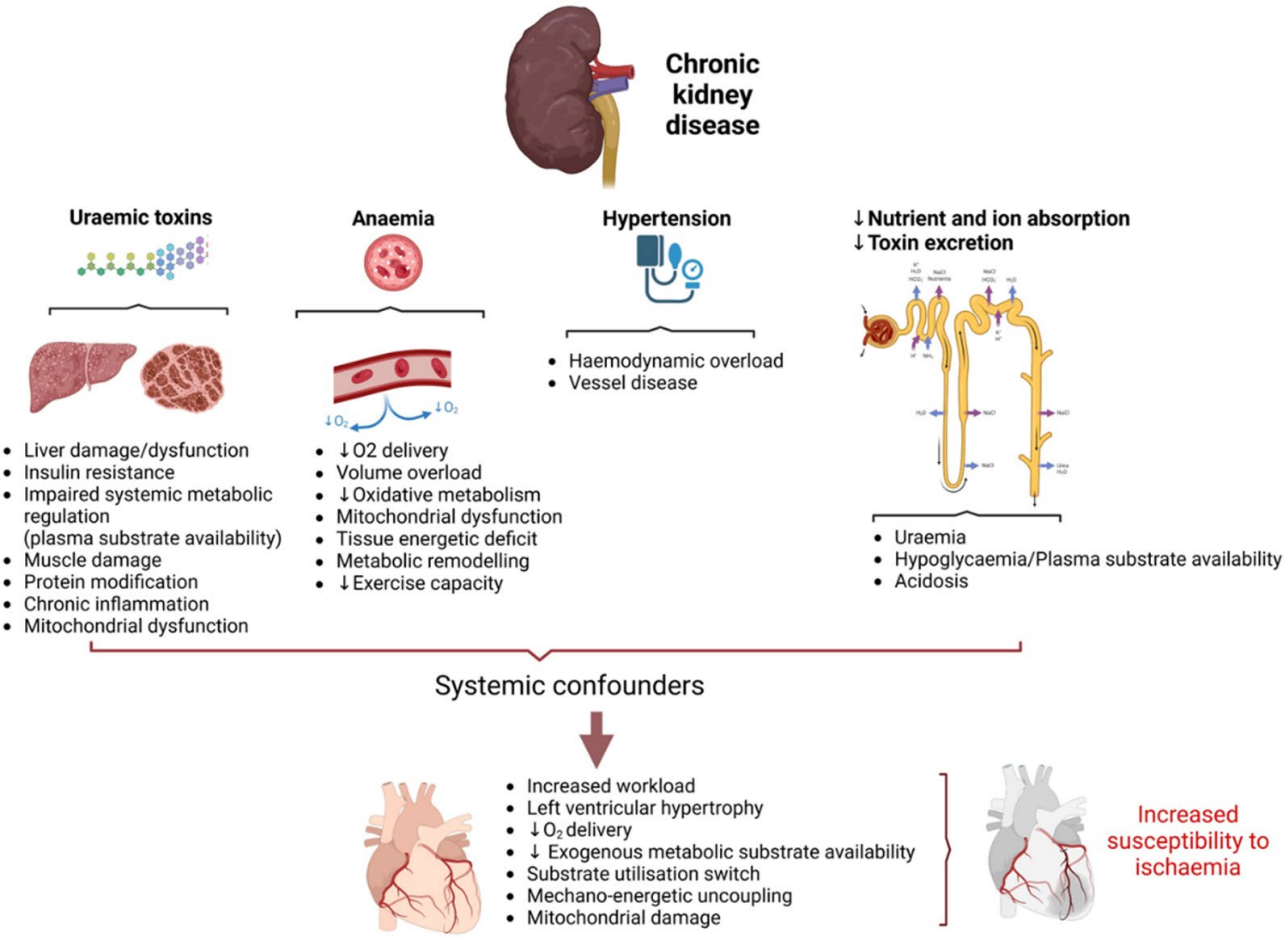
The impact of the systemic confounders on cardiac outcomes in CKD—mechanistic summary. Figure created in www.biorender.com.

How do systemic metabolic confounders common to both models predispose to poor cardiovascular outcomes in CKD? Renal dysfunction is known to lead to accumulation of CKD toxins such as indoxyl sulfate, kynurenic acid, indole-3-acetic acid, p-cresol sulfate which have been shown to directly inhibit mitochondrial oxidative phosphorylation, cause metabolic perturbation and insulin resistance in peripheral tissues^28,34,35^ (Fig. 8). The association between uremic toxins and mitochondrial dysfunction is increasingly recognized as an important mechanism underlying multi-organ impairment in CKD^36^. Indoxyl sulfate and p-cresyl sulfate have been shown to exert direct mitochondrial toxicity in both skeletal muscle and cardiac cells^28,37,38^. Mechanistically, these toxins can impair mitochondrial respiration by disrupting the electron transport chain, particularly at complexes I and III, leading to increased production of reactive oxygen species and oxidative damage^36,37^. Indoxyl sulfate, in particular, has been shown to suppress PGC-1α expression, thereby limiting mitochondrial biogenesis and reducing the cell’s capacity for oxidative metabolism^37^. Moreover, these toxins can activate pro-inflammatory and profibrotic pathways through engagement of the aryl hydrocarbon receptor (AhR), contributing to mitochondrial damage and cellular dysfunction^38^. Recent studies using in vitro and in vivo models have reinforced the causal relationship between uremic toxin exposure and impaired mitochondrial dynamics, including imbalanced fission/fusion, mitophagy inhibition, and reduced ATP production. Together, these findings support the hypothesis that uremic toxins contribute to systemic bioenergetic failure in CKD by targeting mitochondrial integrity and function, providing a mechanistic link between renal dysfunction and peripheral organ injury.

Moreover, severely decreased haematocrit (anaemia) impairs O_2_ delivery resulting in organ hypoxia^39^ further exacerbating metabolic remodelling, mitochondrial dysfunction thus leading to bioenergetic deficit (Fig. 8). Loss of glucose through impaired renal reabsorption also leads to reduced plasma availability. Collectively, this perfect ‘storm’ of events associated with CKD impacts systemic metabolism, triggers organ dysfunction and predisposes hearts to remodelling (Fig. 8).

Our findings suggest that systemic bioenergetic impairment may contribute to both skeletal muscle dysfunction and cardiac vulnerability in CKD. Several metabolic pathways may offer therapeutic potential to improve muscle energetics and mitigate cardiac decline. Agents that activate AMP-activated protein kinase (AMPK), such as metformin or AICAR, may enhance mitochondrial biogenesis and oxidative metabolism^40^. PPARα agonists like fenofibrate have been shown to promote fatty acid oxidation and reduce lipid accumulation, which may alleviate lipotoxic stress in both muscle and cardiac tissue. SGLT2 inhibitors (i.e. empagliflozin) have also demonstrated favourable effects on metabolic efficiency and mitochondrial function in both preclinical and clinical studies, independent of their glycemic control. Additionally, mTOR inhibitors (i.e. rapamycin) and SIRT1 activators may help restore nutrient sensing and improve metabolic flexibility. Finally, strategies aimed at reducing uremic toxin levels, such as AST-120, may help preserve mitochondrial integrity in peripheral tissues^37,41,42^. These pharmacological approaches warrant further investigation as potential interventions to support systemic energy metabolism and protect against CKD-associated cardiac dysfunction.

Crosstalk between organs has been identified in HFpEF of different aetiologies^43^ and likely contributes to the systemic nature of CKD including the development of CKD cardiomyopathy. Based on our findings, we can hypothesize that the systemic metabolic alterations observed in CKD contribute causally to muscle and cardiac dysfunction, rather than being solely a consequence of tissue injury. For instance, alterations in circulating metabolic milleau precede or occur concurrently with early markers of myocardial remodeling and dysfunction. However, the potential role of uremic toxins, chronic inflammation, and other CKD-associated systemic stressors in driving both metabolic dysregulation and organ injury cannot be fully excluded. Thus, we propose a bidirectional model: systemic metabolic disturbances both contribute to and result from CKD-induced tissue damage. Further mechanistic studies are warranted to disentangle these complex interactions and establish causality. Nevertheless, improved understanding of organ interplay can enhance monitoring protocols and pave the way for interventions that can mitigate the adverse effects of CKD on overall metabolic health. Moreover, therapeutic targeting and prevention of systemic metabolic derangement may be a new approach to ameliorate aberrant cardiac remodelling in CKD.

### Study limitations

While our study provides valuable insights into metabolic alterations in CKD, we acknowledge that direct evaluation of mitochondrial homoeostasis in the rat models was not performed. Incorporating targeted assessments of mitochondrial function (i.e. isolated mitochondrial respiration, electron transport chain protein expression, mitochondrial DNA content) in future preclinical studies would further strengthen our understanding of the underlying mechanisms. We acknowledge that only male rats were used in this study, which may limit the broader applicability of our findings, particularly given reported sex-related differences in cardiac and skeletal muscle responses to CKD. Future studies including female animals will be important to fully understand potential sex-specific effects. While the adenine and PN models capture distinct pathological features of CKD (interstitial fibrosis and glomerulosclerosis, respectively) they do not fully recapitulate the heterogeneity of human CKD, which often involves a complex interplay of comorbidities and variable disease progression. Additionally, although we assessed skeletal muscle bioenergetic capacity in human CKD patients using ^31^P-MRS, a direct comparison with cardiac bioenergetic reserve is limited by the lack of equivalent assessment in human hearts. Future studies employing ^31^P NMRs or advanced imaging techniques (PET, MRI, echocardiography-based measures of mechanical efficiency) may help bridge this gap.

## Supplementary Information

Below is the link to the electronic supplementary material.

Supplementary Material 1

Supplementary Material 2

## Data Availability

The datasets generated during the current study are provided in the data supplement file.
